# Neutrophils in idiopathic pulmonary fibrosis patients are phenotypically distinct from controls

**DOI:** 10.1183/23120541.00424-2025

**Published:** 2025-12-22

**Authors:** Deborah L.W. Chong, Jagdeep Sahota, Emma K. Denneny, Theresia A. Mikolasch, Helen S. Garthwaite, Melissa Heightman, Helen Booth, Joanna C. Porter

**Affiliations:** 1 Department of Medicine, City St George's, University of London, London, UK; 2 UCL Respiratory, University College London, London, UK; 3 Thoracic Medicine, University College London Hospital, London, UK

## Abstract

Neutrophils, key immune cells, are increased in the air-spaces of patients with idiopathic pulmonary fibrosis (IPF) and serve as an independent prognostic biomarker [1, 2]. Recent studies show that blood neutrophil-to-lymphocyte ratios correlate with IPF lung function and predict outcome [3]. Neutrophils may contribute to fibrotic lung disease *via* the production of reactive oxygen species (ROS), proteinases (*e.g.* neutrophil elastase), and neutrophil extracellular traps [4–7].

## Shareable abstract

**Neutrophils in IPF patients exhibit functional distinctions, suggesting a mechanism for their recruitment into the lungs. This study underscores the need for further research into the role of neutrophils in the progression of IPF.** https://bit.ly/4ldKo9b

*To the Editor:*

Neutrophils, key immune cells, are increased in the air-spaces of patients with idiopathic pulmonary fibrosis (IPF) and serve as an independent prognostic biomarker [1, 2]. Recent studies show that blood neutrophil-to-lymphocyte ratios correlate with IPF lung function and predict outcome [3]. Neutrophils may contribute to fibrotic lung disease *via* the production of reactive oxygen species (ROS), proteinases (*e.g.* neutrophil elastase), and neutrophil extracellular traps [4–7]. However, the precise mechanisms by which neutrophils are recruited and retained within the lung to drive IPF disease progression remain unclear.

Transcriptomic studies highlight that neutrophils are a heterogenous population, with distinct functional subsets occurring during disease [8, 9]. However, no consensus exists regarding specific surface markers for neutrophil sub-types. A subset of mature neutrophils, defined as CD16^bri^CD62L^dim^CD11b^+^, has been demonstrated to have enhanced ROS production, whilst suppressing T-cell responses [10]. Whether neutrophil functionality or surface marker expression is altered in IPF is unknown.

In this observational study, we investigated neutrophil functionality and surface marker expression in a small cohort of patients with IPF (n=5–26) and asymptomatic non-interstitial lung disease (non-ILD) controls (n=4–16) that were consecutively recruited at the national ILD Service at University College London Hospital between May 2017 and January 2020. Written informed consent was obtained from all participants (UK National Health Service Integrated Research Application System study ID 29531). All IPF patients were diagnosed based on clinico-radiological multidisciplinary team review. Non-ILD controls included individuals with haemoptysis (31%), COPD/bronchiectasis (32%), abnormal chest radiographs (25%), asthma (6%) or emphysema (6%). The percentage of non-ILD peripheral neutrophils was comparable with healthy controls (data not shown), to indicate these non-ILD controls did not have systemic inflammation at time of recruitment. In the non-ILD cohort, the mean±sd age was 64±13 years, 50% male, with mean±sd neutrophil blood count of 4.94±2.13 ×10^9^  cells·L^−1^. Patients with IPF were significantly older compared to the non-ILD cohort at 76±8 years (p=0.001), and predominantly male (80%) with neutrophil blood count of 5.94±2.03 ×10^9^ cells·L^−1^, which was not significantly different compared to non-ILD controls (p=0.17).

To investigate neutrophil functionality, blood and bronchoalveolar lavage fluid (BALF) were collected from patients, and neutrophils isolated for neutrophil chemotaxis studies as described [2]. To assess surface marker expression on isolated peripheral blood or BALF neutrophils, flow cytometric analysis (figure 1a) was performed using anti-CD11c-FITC, anti-CD54-APC, anti-CD11b-V450, anti-CD18-FITC, anti-CD16-APC-Cy7 and anti-CD62L-PE fluorescently conjugated antibodies (all from BD Biosciences, Wokingham, UK) in separate cohorts of patients with IPF or non-ILD controls. In later experiments, an anti-CXCR1-PerCP-Cy5.5 antibody (BD Biosciences) was added to the staining panel. Flow cytometric data were analysed using FlowJo v10.10 software (BD Biosciences). Two-way ANOVA was performed, with Holm–Šidák multiple comparisons *post hoc* testing, using GraphPad Prism v10.4.1 software (GraphPad, Boston, MA, USA).

**FIGURE 1:**
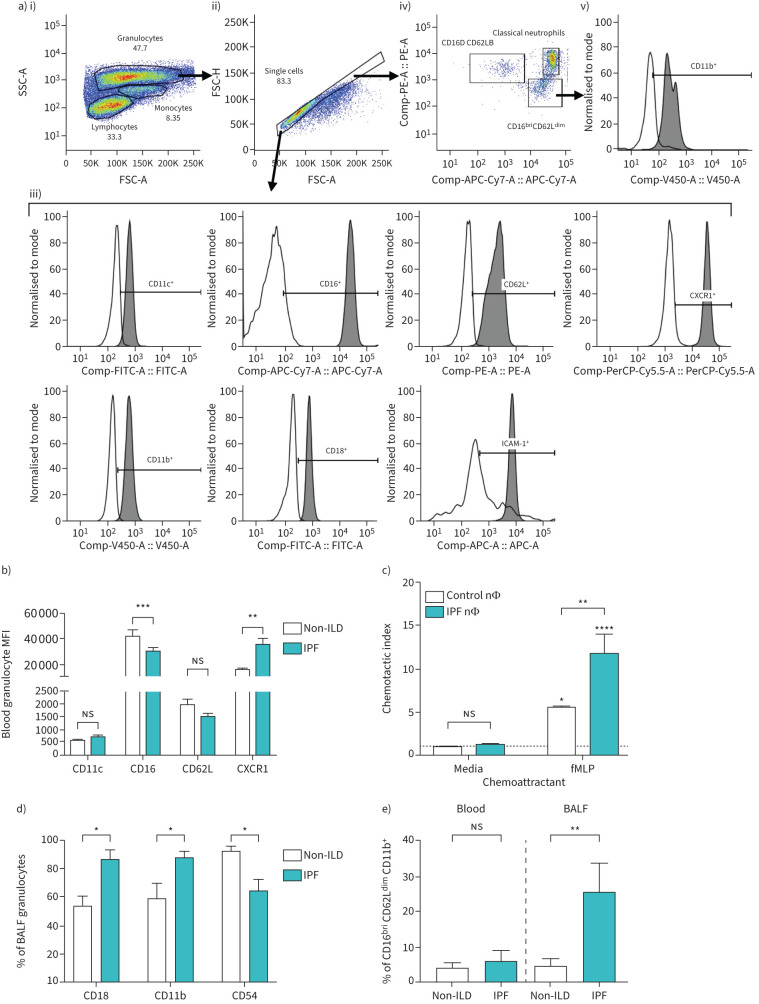
Neutrophils from idiopathic pulmonary fibrosis (IPF) patients exhibit phenotypic and functional differences compared to non-interstitial lung disease (non-ILD) controls. a) Representative flow cytometric gating strategy assessing surface marker expression on peripheral or bronchoalveolar lavage fluid (BALF) neutrophils from IPF or non-ILD control patients. Granulocytes were identified based on i) forward scatter (FSC) *versus* side scatter (SSC) parameters, and ii) doublet exclusion. iii) Expression of CD11c, CD16, CD62L, CXCR1, CD11b, CD18 and CD54 (ICAM-1) on gated neutrophils (shaded histograms) was defined based on isotype control staining (unshaded histograms). iv) Expression of CD16 *versus* CD62L was investigated on neutrophils, along with v) CD11b expression on the CD16^bri^CD62L^dim^subset. b) Median fluorescence intensity (MFI) of CD11c, CD16, CD62L or CXCR1 expression on peripheral blood granulocytes from IPF (n=5–24) or non-ILD (n=4–15) patients. c) Chemotaxis index of peripheral blood neutrophils (nΦ) from IPF (n=7) or non-ILD control (n=6) patients towards *N*-formyl methionyl-leucyl-phenylalanine (fMLP) (100 nM), as normalised to media controls. d) Percentage of BALF neutrophils expressing CD18, CD11b or CD54 (ICAM-1) from IPF (n=6) or non-ILD (n=7) patients. e) Percentage of CD16^bri^CD62L^dim^CD11b^+^ neutrophils of total granulocytes in peripheral blood or BALF from IPF (n=26 and 6 respectively) or non-ILD (n=16 and 8 respectively) patients. Two-way ANOVA was performed, with Holm–Šidák *post hoc* testing. ns: nonsignificant; *: p<0.05; **: p<0.01; ***: p<0.001; ****: p<0.0001.

Flow cytometric analysis revealed that peripheral neutrophils from IPF (n=24) and non-ILD control patients (n=15) have equivalent expression of CD11c, a neutrophil maturation marker (figure 1b, p=0.987), suggesting unaltered maturation. However, expression of CD16, another neutrophil maturation marker, was significantly reduced on peripheral IPF neutrophils compared to non-ILD controls (figure 1b, p=0.001). Expression of CD62L, a cell adhesion molecule, was comparable between the two patient cohorts (figure 1b, p=0.986). Furthermore, we found that CXCR1, a chemokine receptor, was significantly upregulated in IPF neutrophils compared to controls (figure 1b, 35 305±10 348 *versus* 15 122±3067 MFI (median fluorescence intensity) units respectively, p=0.005). IPF neutrophils also showed a two-fold increase in chemotaxis towards *N*-formyl methionyl-leucyl-phenylalanine (fMLP), a well-described neutrophil chemoattractant, compared to controls (figure 1c, p=0.002). It is known that increased secretion of neutrophil chemokines (including CXCL8, which has high affinity for CXCR1) is found in the lungs and airways of patients with IPF compared to controls [11]. Therefore, increased expression of CXCR1 on peripheral neutrophils may allow for increased trafficking towards chemokines expressed in the lung during IPF.

Patients with IPF have significantly more neutrophils in their BALF than non-ILD controls (44.7±12.5% *versus* 22.4±8.2% of total cells respectively, p=0.016). Further analysis also showed an increased frequency of BALF neutrophils expressing more CD18 (86.6±14.4% *versus* 53.9±16.9% respectively, p=0.014; 3344±1894 *versus* 1934±1058 MFI units respectively) and CD11b (87.9±9.9% *versus* 60.0±27.0% respectively, p=0.012; 2121±1402 *versus* 1402±363 MFI units respectively) in IPF compared to non-ILD controls (figure 1d). Additionally, fewer IPF BALF neutrophils expressed CD54 (ICAM-1), a marker for reverse transmigration, than control neutrophils (figure 1d, p=0.042, 4226±2911 *versus* 21 486±12 123 MFI units respectively). We also found a trend of decreased ICAM-1 expression on peripheral neutrophils from IPF patients compared to non-ILD controls, but this did not reach statistical significance (p=0.205, data not shown). Reverse transmigration is a process by which neutrophils exit inflamed tissues and return back into the circulation for removal by apoptosis in the bone marrow and is characterised by high ICAM-1 expression [12]. Our data suggest that neutrophils in IPF patients may be retained in the lung and are not reverse transmigrating back into the circulation.

Lastly, given that CD16 expression was significantly altered on peripheral neutrophils from IPF patients, we explored the expression of other surface markers, CD62L and CD11b, on CD16 expressing neutrophils. Whilst the percentage of CD16^bri^CD62L^dim^CD11b^+^ neutrophils was similar in the blood of patients with IPF compared to controls (figure 1e, 5.9±16.1% *versus* 4.2±5.1% respectively, p=0.934), there was a significantly increased frequency of CD16^bri^CD62L^dim^CD11b^+^ neutrophils in the BALF of patients with IPF compared to controls (figure 1e, 25.4±22.4% *versus* 4.6±6.5% respectively, p=0.008). This indicates that neutrophils have differential surface marker expression patterns in different cellular compartments of IPF patients.

Overall, our findings demonstrate that neutrophils from patients with IPF exhibit distinct phenotypic and functional characteristics compared to non-ILD controls, with increased expression of adhesion molecules, enhanced chemotactic responses towards fMLP and reduced ICAM-1 expression. This suggests that in IPF, there may be impaired clearance of neutrophils from the lung. However, more studies are needed to explore expression of FPR1 (the receptor for fMLP) and chemotaxis towards IL-8 (the ligand for CXCR1) in IPF neutrophils. It is also still unclear whether enhanced neutrophil retention in the lung drives fibrotic tissue damage. Furthermore, the increase in BALF CD16^bri^CD62L^dim^CD11b^+^ neutrophils in IPF patients, a subset associated with enhanced ROS production, highlights the need for further research into their role in driving fibrotic disease progression.

This study shows that neutrophils can adopt different phenotypic traits during lung disease. Studies in other chronic lung diseases indicate increased circulating numbers of polymorphonuclear myeloid-derived suppressor cells, which correlate with poor outcome in patients with lung cancer [13]. Moreover, aged neutrophils, defined as CD11b^hi^CD62L^lo^CXCR4^+^, are associated with increased neutrophil extracellular trap production and fibrotic disease progression in a murine model [14, 15]. Further research will elucidate the specific mechanistic contributions of neutrophil subsets in IPF and whether they correlate with disease outcome.

This study does have some limitations, including small patient sample size. Additionally, our non-ILD control group consisted of a heterogeneous group of patients, some of whom have chronic lung diseases, associated with neutrophil-driven inflammation. However, it was not possible to access BALF from healthy controls as a comparator group to study neutrophil phenotype under our study ethics. Furthermore, despite including patients with other respiratory diseases, we still found differences between IPF and non-ILD controls and this may be due to different neutrophil function in these chronic lung diseases (*e.g.* inflammatory *versus* pro-fibrotic).

In summary, our findings indicate that neutrophil phenotype and function are modulated in IPF, and suggest a potential mechanism for their role in fibrotic disease progression. Further detailed exploration into the role of neutrophils in fibrotic lung disease may pave the way for the development of novel therapeutic strategies to mitigate neutrophil-driven fibrotic lung damage.
